# The Reality of Antimicrobial Resistance and Antibiotic Usage Data in Asia: The CAPTURA Experience

**DOI:** 10.1093/cid/ciad580

**Published:** 2023-12-20

**Authors:** Toby Leslie, Claudia Parry, Pascale Ondoa, Timothy Walsh, Catrin Moore, Nimesh Poudyal, Florian Marks, N Claire Gordon

**Affiliations:** Fleming Fund Management Agent, Mott MacDonald, London, United Kingdom; Fleming Fund Management Agent, Mott MacDonald, London, United Kingdom; African Society for Laboratory Medicine, Addis Ababa, Ethiopia; Department of Global Health, University of Amsterdam, Amsterdam Institute for Global Health and Development, Amsterdam, The Netherlands; Department of Biology, University of Oxford/Ineos Oxford Institute for Antimicrobial Research, Oxford, United Kingdom; Institute of Infection and Immunity at St George's, University of London, London, United Kingdom; International Vaccine Institute, Seoul, Republic of Korea; International Vaccine Institute, Seoul, Republic of Korea; Cambridge Institute of Therapeutic Immunology and Infectious Disease, University of Cambridge School of Clinical Medicine, Cambridge, United Kingdom; Heidelberg Institute of Global Health, University of Heidelberg, Heidelberg, Germany; Madagascar Institute for Vaccine Research, University of Antananarivo, Antananarivo, Madagascar; UK Health Security Agency, London, United Kingdom

**Keywords:** antimicrobials, antibiotics, resistant, resistance, CAPTURA, Asia, data

## Abstract

Antimicrobial resistance (AMR), particularly in low- and middle-income countries, is threatening to undermine advances in health and development. Scarce technical and human resources in these countries limit the collection of quality AMR data for evidence-based decision-making. The CAPTURA consortium, funded by the Fleming Fund, was implemented across 7 countries in the South and Southeast Asian region. The program focused on collating historical bacteriological data for qualitative and quantitative analyses. The team gathered standard data on the quality of laboratories and clinics and the quality and quantity of retrospective historical AMR data. In addition, retrospective data on antimicrobial use and consumption were analyzed. While standard protocols guided the project, a tailored approach for stakeholder engagement was implemented to work with countries and secure data-sharing agreements. The program also had to navigate the challenges of the COVID-19 pandemic, making some innovative adaptations to overcome logistical barriers. From 2018 through 2022, a large body of data was collected that was used to base a series of recommended key measures for strengthening the development of standardized national surveillance programs and to support alignment with international efforts.

## Teaser

We discuss the background, scope, and outcomes of the Capturing data on Antimicrobial resistance Patterns and Trends in Use in Regions of Asia project, funded by the Fleming Fund and implemented in 7 South and Southeast Asian countries.

Resistance to commonly used antimicrobials used to treat bacterial infections is a current and growing problem throughout the world [1]. Containing antimicrobial resistance (AMR) is recognized as a global health priority to reduce and prevent the socioeconomic costs, mortality, and morbidity that are predicted [2, 3].

Low- and middle-income countries (LMICs) are likely to suffer disproportionately from AMR, having a greater burden of infectious diseases, a greater risk of injudicious use of antimicrobials that results from poor access to healthcare and diagnostics, and limited access to newer antimicrobial agents required to treat resistant infections, each of which can result in longer and more severe illnesses caused by resistant bacteria.

To be effective, national control strategies need to be developed based on accurate and context-relevant data. However, the scale and impact of the problem in LMICs is also difficult to quantify: the very same health system weaknesses that are causing AMR also affect a country’s capacity to conduct the systematic quality-assured surveillance that is needed to make informed decisions at both the practice and policy levels.

The lack of data from LMICs is evident in reports from the World Health Organization Global Antimicrobial Resistance and Use Surveillance System (GLASS), which aims to standardize reporting on AMR and antimicrobial use (AMU) across member states [4]. Currently, the proportion of data submitted is heavily skewed toward higher-income countries, leaving critical gaps in global surveillance data [5]. The majority of LMICs have not established sufficiently robust AMR surveillance systems nationally, which explains the low reporting to GLASS and lack of ability to inform national policy, practice, and research agendas. Even fewer data are available on the use and consumption of antibiotics, where the majority of the data are from high-income countries.

The Fleming Fund is a UK government initiative that was established in 2015 specifically to address these gaps. The Fleming Fund grants program supports the development of national surveillance systems in LMICs, with a specific focus on national data collection, analysis and use, and participation in the GLASS program. In order to improve understanding of temporal changes in AMR, the Fleming Fund has funded retrospective data collection through the Capturing data on Antimicrobial resistance Patterns and Trends in Use in Regions of Asia (CAPTURA). The consortium was commissioned to examine the quality and quantity of historical bacteriological data in LMICs in South and Southeast Asia and to collect metadata on the location and capabilities of microbiological laboratories and biorepositories. The scope of the project also allowed for an extensive assessment of the AMR landscape in the region, including national AMR control programs, country capabilities for generating data, national surveillance networks, and national use of data in planning and advocacy.

In this supplement, the CAPTURA consortium presents a collection of articles that illustrate the scale of the project, describe the methodological factors and uses of laboratory metadata and microbiology data, and examine the extent and quality of antibiotic use/consumption data.

Central to the success of the program was engagement with stakeholders at the country level, allowing the program to succeed even during the COVID-19 pandemic, which was facilitated by having a national lead based in each country. The program worked closely with national surveillance personnel and institutions (such as AMR coordinating committees and technical working groups) to promote country ownership.

The program also indirectly tested the willingness of countries to share data with external collaborators. Global data sharing is a prerequisite to participation in GLASS, and it is vital that the countries recognize the importance and value of their data at the international level. While 10 of the 12 countries showed a willingness to engage, only 7 countries ultimately granted permission for sharing data with the consortium, despite the offer and use of negotiated data-sharing agreements. Although country stakeholders are generally aware of the importance and benefits of sharing AMR data, the lack of clear data access policies and regulations (at the national and regional levels) created uncertainties about data ownership and concerns about access and use of the data [6], therefore hindering data-sharing efforts. South Asian countries were far more likely to share data than countries in Southeast Asia (98% of the data originated from South Asia, and only 2% from countries in Southeast Asia), highlighting a major challenge faced by international surveillance programs. Notably, several countries whose data are presented in this supplement engaged strongly with this process and are to be applauded for their proactive approach to the global problem of AMR. It is no coincidence that these countries are the same ones that are showing the best progress toward addressing AMR.

As well as AMR data, the program collected retrospective data on national-level antimicrobial consumption via information on total sales, import/export quantities, records on antimicrobial procurement, and supply and/or distribution at the district or facility level. This information is key to understanding the overall quantities of antimicrobials used nationally, as well as giving indicative information regarding the distribution between different sectors. Part of the strength of the program was collecting these complementary data that had never been collated before.

An important part of the approach was to use a standard set of variables to rate the quality of laboratory and clinical data collected at each site. This allowed comparisons between the countries and identified important gaps. One of the most important findings is that there was little standardization in the data. Very few sites routinely collected basic laboratory, clinical, and treatment history information, and there was limited national agreement regarding the choice of antimicrobials tested against particular pathogens. The program also noted the need to improve the collection and use of data on AMU at the hospital level, in particular, linking prescription data with patient clinical information.

The COVID-19 pandemic had a significant impact on the implementation of the program. While the global shutdowns resulted in many logistical difficulties, the consortium adapted by identifying 4 streams of activities that could be supported via online platforms and therefore could continue regardless. These included stakeholder engagement, capacity building, mapping of relevant facilities, and (to some degree) data collection. While each country required a tailored approach, the results demonstrate the feasibility of program implementation even in the face of a pandemic. Lessons learned may inform future similar programs operating under more normal conditions. The need to reduce monetary and carbon costs means that innovative ways of providing international support to countries while minimizing the impact on program effectiveness are to be welcomed.

The consortium made a series of recommendations for improving and expanding surveillance systems based on the 4 years of experience and data collection. These include using a standard data format with a minimum acceptable set of key variables, ensuring access to relevant tools, improving digitization of data, and improving the collection of key clinical information such as diagnosis and treatment alongside isolate, susceptibility, and prescribing data. Many of these recommendations will be the focus of ongoing work by the consortium and the Fleming Fund as a whole.

The findings support the need for standardized national surveillance programs that are aligned with international efforts such as GLASS. These will require continued resources, political commitment, and international collaboration to ensure implementation and sustainability.

## Notes

***Disclaimer.*** The views expressed here are those of the authors and not necessarily those of the UK Department of Health and Social Care or its management agent, Mott MacDonald; and, International Vaccine Institute.

***Supplement sponsorship.*** This article appears as part of the supplement “Capturing Data on Antimicrobial Resistance Patterns and Trends in Use in Regions of Asia (CAPTURA),” funded by the Department of Health and Social Care's Fleming Fund using UK aid.

***Financial support*.** This work was supported by the Fleming Fund regional grants (grant numbers FF10-135 and FF11-139). The Fleming Fund is a UK aid investment to support surveillance of antimicrobial resistance in low- and middle-income countries in Africa and Asia. The regional grants program is managed by Mott MacDonald Ltd, the Fleming Fund Grants Management Agent.
